# Estimation of Chlorophyll-a Concentration in Turbid Lake Using Spectral Smoothing and Derivative Analysis

**DOI:** 10.3390/ijerph10072979

**Published:** 2013-07-16

**Authors:** Chunmei Cheng, Yuchun Wei, Xiaopeng Sun, Yu Zhou

**Affiliations:** Key Lab of Virtual Geographic Environment, Ministry of Education, Nanjing Normal University, Nanjing 210023, China; E-Mails: ccm8711@163.com (C.C.); sunxiaopeng1226@gmail.com (X.S.); redevil7@126.com (Y.Z.)

**Keywords:** chlorophyll-a, first-order derivative, hyperspectral reflectance, remote sensing, Taihu Lake

## Abstract

As a major indicator of lake eutrophication that is harmful to human health, the chlorophyll-a concentration (Chl-a) is often estimated using remote sensing, and one method often used is the spectral derivative algorithm. Direct derivative processing may magnify the noise, thus making spectral smoothing necessary. This study aims to use spectral smoothing as a pretreatment and to test the applicability of the spectral derivative algorithm for Chl-a estimation in Taihu Lake, China, based on the *in situ* hyperspectral reflectance. Data from July–August of 2004 were used to build the model, and data from July–August of 2005 and March of 2011 were used to validate the model, with Chl-a ranges of 5.0–156.0 mg/m^3^, 4.0–98.0 mg/m^3^ and 11.4–35.8 mg/m^3^, respectively. The derivative model was first used and then compared with the band ratio, three-band and four-band models. The results show that the first-order derivative model at 699 nm had satisfactory accuracy (R^2 ^= 0.75) after kernel regression smoothing and had smaller validation root mean square errors of 15.21 mg/m^3^ in 2005 and 5.85 mg/m^3^ in 2011. The distribution map of Chl-a in Taihu Lake based on the HJ1/HSI image showed the actualdistribution trend, indicating that the first-order derivative model after spectral smoothing can be used for Chl-a estimation in turbid lake.

## 1. Introduction

Freshwater lakes are the main source of drinking and agricultural water in many areas, and their water quality can greatly affect human health. Due to the increasing economic development in China, lake eutrophication has become a serious water quality problem that has recently attracted much attention. The main danger caused by eutrophication is the toxins produced by some algae, which are harmful for drinking and can poison or even kill humans and animals that consume contaminated water and food [1].

The chlorophyll-a concentration (hereafter referred to as Chl-a) is an important parameter in evaluating water quality, nutrition status and organic pollution extent, providing useful information for managing water quality [2] and monitoring water pollution [3]. Compared with monitoring Chl-a through field water sampling, which is usually costly and time-consuming, remote sensing is a robust and effective means of monitoring large areas of lake water and is widely used in the assessment of Chl-a and trophic conditions in lakes.

*In situ* spectra above the water surface obtained through a spectrometer can indicate the optically active material in water and be used as the important raw data for the remote sensing estimation model of Chl-a. The hyperspectral reflectance of turbid lake water is an expression of the compound information of the water components, including chlorophyll-a, suspended sediment and colored dissolved organic matter (CDOM) [4] and is often affected by certain factors, such as the measuring instruments and environment conditions. Therefore, the preprocessing of the spectrum is of great importance for extracting better information about Chl-a.

The derivative analysis of spectra is effective for information detection [5] and has already been commonly applied in analytical chemistry [6]. Spectral derivatives potentially allow for the elimination of background signals and resolution of overlapping spectral features [7]. Derivatives of a second or higher order are relatively insensitive to variations in illumination intensity caused by changes in the sun angle, cloud cover and topography. For example, fourth-derivative spectroscopic analysis was applied to phytoplankton absorption spectra to determine Chl-a and phytoplankton pigments that could be identified as chemotaxonomic markers [8].

In addition to laboratory spectroscopic analysis, the derivative method can also be used for tackling analogous problems, such as interference from water and other background in the remote sensing retrieval of Chl-a in water. Previous studies showed that the first-order derivative is able to remove pure water effects and the second-order derivative can remove suspended sediment effects [9]. Han [10] demonstrated that the first-order derivative spectrum at 690 nm can be used for Chl-a estimation in the presence of other water constituents and that its performance is better than the traditional band ratio model. Han [11] and Chen [12] used a first-order derivative model for chlorophyll inversion, and Shi [13] used the second-order derivative model to retrieve Chl-a, both of which achieved better precision. Duan [14] compared several semi-empirical algorithms for Chl-a inversion and found that the first-order derivative model had a higher accuracy than the band ratio and three-band model. Huang [15] made a similar comparison, and the calculations showed that the first-order derivative model was better than the single-band and band ratio model in the Chl-a estimation of Tangxun Lake, China.

However, derivatives are notoriously sensitive to noise, and direct spectral derivative processing will magnify the noise. Therefore, smoothing or otherwise minimizing random noise is necessary. Tsai [16] reviewed and modified several smoothing and derivative computation algorithms to develop a set of cross-platform spectral analysis tools for applying derivative spectral analysis to remote sensing data, pronouncing that the mean-filter smoothing algorithm is an appropriate pretreatment prior to the derivative computation. There are some other commonly used smoothing methods in addition to the “mean-filter algorithm”, such as “Savitzky-Golay polynomial smoothing” and the “kernel regression algorithm”, and all of them are compared in this study, which aims to identify the most appropriate smoothing method. The mean-filter algorithm uses a specific mean filter to discriminate between signal and noise, and Savitzky-Golay smoothing uses polynomial and least squares fitting to determine the signal within the moving window; both are dependent on the window width [17]. Kernel regression smoothing has proven to be a recommended smoothing method to enhance the signal in the spectrum above the water surface because it can remove the normal distribution interference of the spectrum [18].

The objectives of this study are the following: (1) to compare the influence of three typical smoothing methods on the spectrum and select the most suitable smoothing method; (2) to build a derivative model based on the *in situ* spectra from 2004 after spectral smoothing, validate its performance based on the data from 2005 and 2011 and compare the results with those of the band ratio, three-band and four-band models; and (3) to generate a Chl-a distribution map of Taihu Lake based on the hyperspectral image of China’s HJ1 satellite.

## 2. Data and Methods

### 2.1. Study Area and Data Collection

Taihu Lake, the second largest freshwater lake in China and with typically turbid water, is located at the junction of Jiangsu and Zhejiang provinces. Taihu Lake covers an area of 2,427.8 km^2^ and has an average depth of 2.12 m, with eutrophication status ranging from moderate to heavy [19].

**Figure 1:**
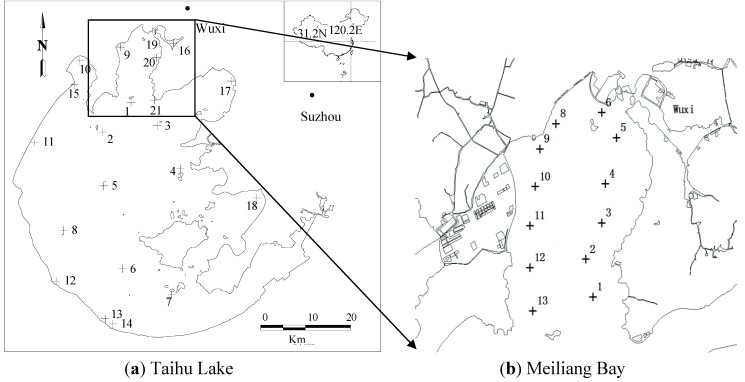
Sample distribution of Taihu Lake in (**a**) July-August of 2004 and 2005 and (**b**) March of 2011.

In July–August of 2004 and 2005, water samples covering the lake were collected, and the spectrum above the water surface was measured at Taihu monitoring sites. In March 2011, the sampling positions mainly covered the Meiliang Bay heavy eutrophication area. The *in situ* spectra were collected and the water samples were refrigerated until the laboratory analysis within 24 h. The sample distribution of the three datasets is shown in Figure 1, and the sample numbers in 2004, 2005 and 2011 were 23, 21 and 12, respectively, after discarding the samples under windy or cloudy conditions.

The *in situ* spectrum was measured using the ASD Field Pro Spectroradiometer (Analytical Spectral Devices Inc., Boulder, CO, USA), with a spectral range of 350–1,050 nm. The field of view angle was 10°, and the spectrum was measured at 0.5–1 m above the water surface. The observation geometry was based on that of Mueller [20]. The spectral reflectance curve from 400 to 900 nm was used to calculate the remote sensing reflectance, and other wavelength parts were discarded due to noise. The reflectance of the standard gray plate was 30%. The median of the repeated measurements was selected as the sample reflectance to build the model.

Chl-a was analyzed in the laboratory according to national standard SL88-1994 in China (three-color spectrophotometry). First, the water samples collected in the field were filtered through a GF/C membrane. The membrane was dried in a low-temperature refrigerator for more than 12 h and then the chlorophyll was extracted by 90% acetone from the filter. The extracted liquid was centrifuged and kept still for 12 h, and the supernatant was then measured in a UV-2550 spectrophotometer. The Chl-a was calculated using the absorbance at 750 nm, 663 nm, 645 nm and 630 nm.

The statistical characteristics of Chl-a in the three datasets are shown in Table 1. The sample with the highest Chl-a (192 mg/m^3^) in 2004 was not used because its spectrum is similar to that of algae bloom.

**Table 1:** Statistical characteristics of Chl-a in July–August of 2004 and 2005 and in March of 2011.

Dataset	Sample numbers	Minimum	Maximum	Median
2004	23	5.0	156.0	33.0
2005	21	4.0	98.0	29.0
2011	12	11.4	35.8	23.3

The total suspended sediment concentration (TSS) was measured according to the gravimetric method (China national standard GB11901-89, 1990). Based on the measurements from 1998 to 2003 in Taihu Lake, the average TSS value in July–August was 49.2 mg/L, ranging from 12.0 mg/L to 261.0 mg/L. The TSS value in Meiliang Bay ranged from 7.3 mg/L to 21.1 mg/L in March of 2011.

### 2.2. Spectral Smoothing and Spectral Derivative

Three typical smoothing methods, *i.e.*, “mean filter”, “Savitzky-Golay polynomial smoothing”, and “kernel regression smoothing”, were used in this study. The first two algorithms were conducted using the “smooth” function from the Matlab toolbox. The kernel regression smoothing algorithm was performed according to Yi Cao [21] using the Gaussian kernel and calculated using the Nadaraya-Watson method [22]. The window width was set as 5 nm first, and all other parameters were set to default [18]. Different window widths were used to smooth the spectrum to find the most suitable smoothing method and window width.

The following formulas were used to calculate the first- and second-order derivatives of remote sensing reflectance:

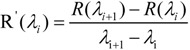
(1)

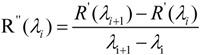
(2)
where *λ*_i+1_, *λ*_i_, and *λ*_i-1_ are the adjacent wavelengths and *R* (*λ*_i_), *R*' (*λ*_i_) and *R*'' (*λ*_i_) are the original spectra, first-order spectral derivative and second-order spectral derivative at band *λ*_i_, respectively.

### 2.3. Model Building and Accuracy Evaluation

The first- and second-order derivative models were built and then compared with other typical models. The band ratio model is commonly used for Chl-a estimation, using the ratio of the reflectance peak near 710 nm to the reflectance valley near 670 nm [23]. The three-band Chl-a estimation model has recently been promoted by Dall’Olmo [24] and Gitelson [25], and it is based on the bio-optical properties of the water body and has been validated in some datasets. Considering the effect of suspended sediment in the near-infrared range, Le [26] proposed a four-band model. The formulas of the three models are as follows:

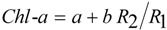
(3)

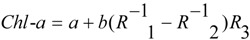
(4)

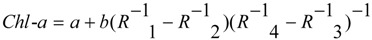
(5)
where a and b are model parameters and *R*_1_, *R*_2_, *R*_3_, and *R*_4_ are the reflectance at the wavelengths of *λ*_1_, *λ*_2_, *λ*_3_ and *λ*_4_, respectively.

With respect to the band ratio model, *λ*_2_ and *λ*_1_ were determined in the range from 700 to 720 nm and 650 to 690 nm, respectively, to minimize the RMSE of *R*_2_/*R*_1_. With respect to the three-band model, the optimal bands were determined in the wavelength range from 450 to 750 nm, with the initial iteration positions of *λ*_1_ and *λ*_3_ at 675 nm and 750 nm, respectively, and the detailed description of the model tuning method was from Zimba and Gitelson [25]. First, the initial positions of *λ*_1_ and *λ*_3_ were used to find the optimal *λ*_2_ position through iteration until the RMSE of the model, (1/R675 − 1/R [*λ*_2_]) × R750, became minimal. Then, *λ*_1_ and *λ*_2_ were fixed to find the position of *λ*_3_ at which the RMSE was minimal. This iterative calculation continued until all three positions no longer changed. The four-band model was built according to the method of Le [26].

The RMSE, average relative error (ARE) and normalized RMSE (NRMSE) were used to evaluate the model accuracy, and their formulas are as follows:

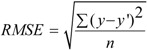
(6)

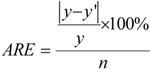
(7)

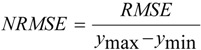
(8)
where *y* is the measured Chl-a (mg/m^3^) , *y*' is the estimated Chl-a (mg/m^3^) , *n* is the sample number, and *y*_max_ and *y*_min_ are the maximum and minimum of measured Chl-a, respectively.

### 2.4. Preprocessing of HJ1/HSI Data

The HJ1 environmental satellite is the first satellite used for environmental monitoring in China, and the hyperspectral imager (HSI) on it has contiguous spectral bands and a short return cycle. The spatial resolution of the HSI data is 100 m, the time resolution is 96 h, and the average spectral resolution is 5 nm, with a total of 115 bands from 450 to 950 nm.

Because the weather in the region around Taihu Lake is perennially cloudy and rainy, obtaining synchronous images with the field data is difficult. The HSI image on 9 May 2009, covering a large part of Taihu Lake, was collected to generate the Chl-a distribution map. The geometric correction was based on a standard TM image with precise geometric information, and the error was within one pixel.

The atmospheric correction of the HJ1/HSI image was conducted using the 6S algorithm, which considered the non-Lambertian surface situation and solved the coupled problems of the BRDF surface and atmosphere [27]. The mid-latitude summer model was selected to be the atmospheric model, and urban aerosol was selected to be the aerosol type of this area. The reflectance of HJ1/HSI image was then calculated according to the correction factors from the 6S model.

## 3. Results and Discussion

### 3.1. Spectral Smoothing

**Figure 2:**
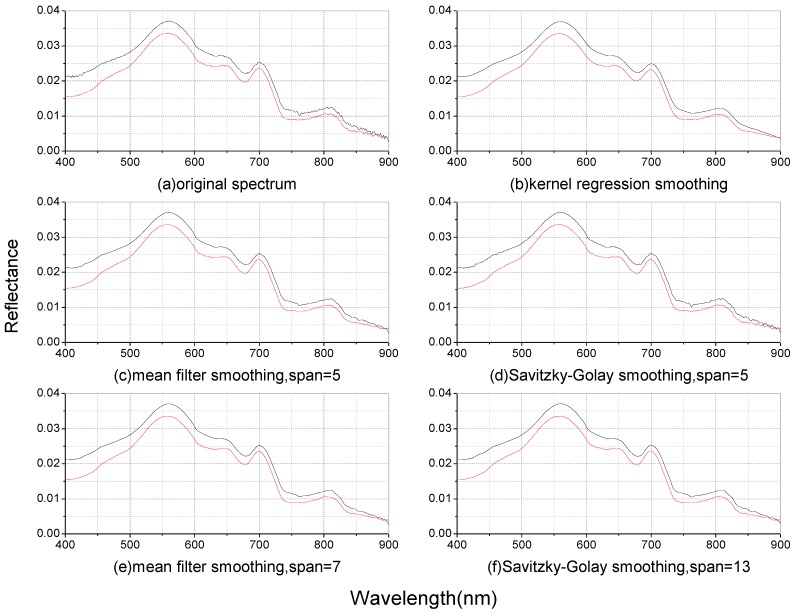
Spectrum reflectance (**a**) of the original spectra, (**b**) after kernel regression smoothing, (**c**, **e**) after mean filter smoothing, and (**d**, **f**) after Savitzky-Golay smoothing.

Spectral derivatives can distinguish the detailed information in the spectrum, but the direct derivative processing of the remote sensing reflectance can magnify the noise caused by the environment or measurement influence. Thus, smoothing the spectrum before derivative calculation is necessary. Three methods were used to smooth two spectra randomly selected from the dataset of 2004, and the spectra before and after smoothing are displayed in Figure 2. No quantitative measurement of the noise was implemented, and the filter size increased gradually until relatively smooth spectra were obtained.

Figure 2 shows the smoothing results of the spectral reflectance. The primary factor controlling the extent of smoothing is the bandwidth of the filter window. In general, a greater filter window size resulted in a smoother result. After using the same filter size of 5 nm, the kernel regression algorithm achieved the best smoothing result, whereas the Savitzky-Golay filter performed no better than the mean-filter algorithm. When gradually increasing the filter size, the mean-filter with a window width of 7 nm and the Savitzky-Golay filter with a window width of 13 nm gave the best results. Previous studies usually used the seven-point mean-filter smoothing algorithm because it is simple and requires the least computation time [10,16].

There is always a trade-off between noise removal and the ability to resolve fine spectral details. As the filter size increases, spectral details may also be suppressed. Ideal smoothing can remove noise without altering the real spectral features, and the optimal filter size for approaching this goal depends on both the noise type and the smoothing algorithm. For the spectra in Figure 2, the mean-filter with a window width of 7 nm or the Savitzky-Golay filter with a window width of 13 nm was required to produce usable derivative spectra. However, because kernel regression smoothing can achieve the best result without much bandwidth searching, kernel regression smoothing was used in this study.

### 3.2. The Chl-a Estimation Model Based on the Spectral Derivative

**Figure 3:**
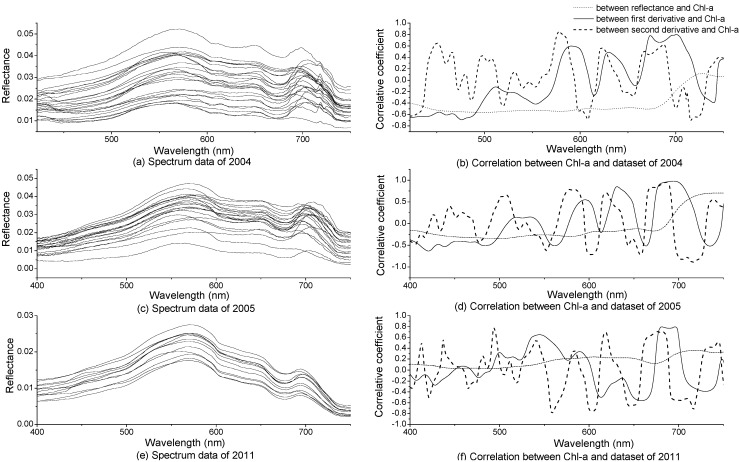
The original spectrum in (**a**) 2004, **(c)** 2005, and (**e**) 2011, and the correlation coefficients between Chl-a and the original reflectance, first-order spectral derivative and second-order spectral derivative in (**b**) 2004, (**d**) 2005, and (**f**) 2011.

The spectrum data from July-August of 2004 and 2005 and March of 2011 were used for the derivative analysis. The magnitude and shape of the reflectance in Figure 3(a,c,e) represent typical turbid water, with a reflectance peak near 580 nm due to the weak absorption of chlorophyll and carotene and to cell scattering. A reflectance valley or shoulder-like feature near 630 nm appears due to the phycocyanin absorption, and a reflectance valley exists near 675 nm due to the strong absorption of chlorophyll a. The reflectance peak at 700 nm may be due to the fluorescence effect of Chl-a [28] or the combined effect of decreasing Chl-a absorption and increasing water absorption [29].

There are some differences in the spectra data of the three datasets: the reflectance peak at 700 nm and valley near 670 nm of the spectra in 2004 and 2005 are obvious, indicating high Chl-a in the phytoplankton-dominated lake water in summer. The fluorescence peak at 700 nm is lower in the spectrum of March 2011 because the suspended solids dominate the water in the winter [30].

All spectra data were smoothed using the kernel regression, and the first- and second-order derivatives were then calculated. The correlation coefficients between Chl-a and the original spectra, first-order spectral derivative, and second-order spectral derivative were calculated and shown in Figure 3(b,d,f).

The spectral derivatives focused mainly on the shape characteristics of the spectral curve while ignoring the magnitude difference and suppressing the background effects from the water and other substances. Thus, the derivatives can accurately extract the spectral characteristics of the water component. Figure 3(b,d,f) show that the correlation coefficients between Chl-a and the first-order spectral derivative of the three datasets had the same characteristics, with a significantly high value near 700 nm. The regularity characteristics of the correlation coefficients between Chl-a and the second-order spectral derivative were not obvious, but a remarkably constant high value near 670 nm could still be observed.

Han [10] demonstrated that the first-order derivative at 690 nm was highly correlated with Chl-a, and Fraser [31] found that the first-order derivatives at 429 nm and 695 nm were correlated significantly with Chl-a. In addition, Shi [13] found that the correlation coefficient between Chl-a and the second-order derivative at 670 nm was much greater than that for the first-order derivative at 700 nm. The results observed in this study that the first-order derivative from 690 to 700 nm and the second-order derivative near 670 nm were highly correlated with Chl-a are consistent with previous studies.

Figure 3(b,d,f) show that the spectral derivative that has a maximum positive correlation with Chl-a is not at a fixed wavelength due to the redshift of the reflectance peak near 700 nm but that the fluctuation is within a relatively stable range. The spectra of first-order derivative that has a maximum correlation with Chl-a generally ranged from 685 nm to 700 nm, and that of the second-order derivative ranged from 670 nm to 685 nm.

**Figure 4:**
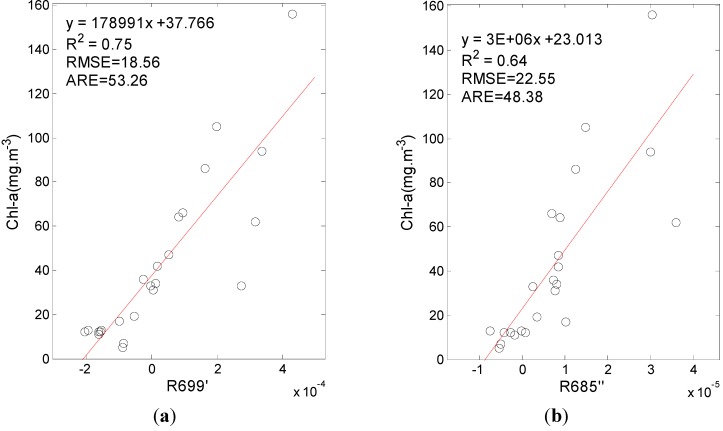
The Chl-a estimation model built using (**a**) the first-order derivative at 699 nm and (**b**) the second-order derivative at 685 nm.

With respect to the spectra data from 2004, the first-order derivative at 699 nm and the second-order derivative at 685 nm were found to be the most highly correlated with Chl-a. Regression models between Chl-a and the first-order derivative at 699 nm and between the second-order derivative at 685 nm were built, and the results are shown in Figure 4.

Figure 4 shows that the results of both the first- and second-order derivative models were acceptable and that the first-order derivative model had a better performance. The Chl-a in 2004 ranged from 5 to 156 mg/m^3^, with the R^2^ of both the first- and second-order derivative models being greater than 0.6 ( *p* < 0.0001) and the RMSEs being less than 22 mg/m^3^. These results also showed that the estimation of lower Chl-a is relatively better than that of high Chl-a because the reflectance peak of high Chl-a redshifts to a longer wavelength, thus decreasing the sensitivity of the derivative spectra to Chl-a.

### 3.3. Model Validation and Model Comparison

**Figure 5:**
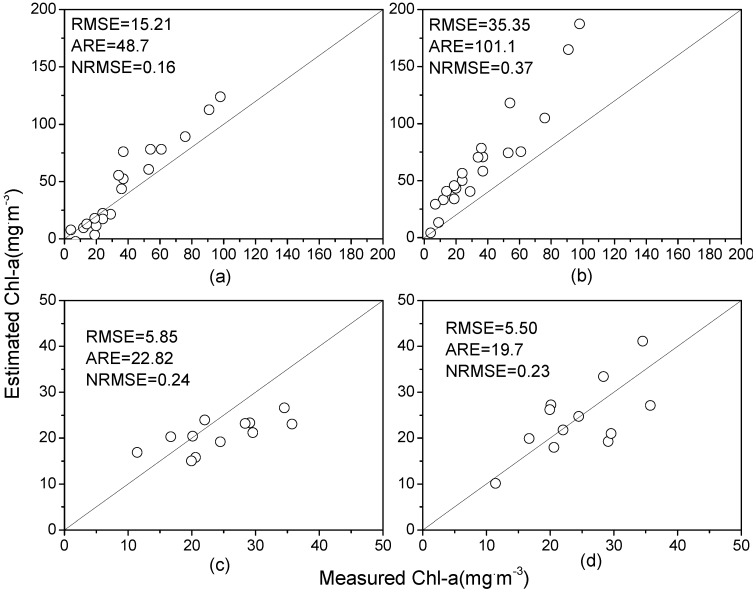
Validation of (**a**) first-order derivative model using data from 2005, (**b**) second-order derivative model using data from 2005, (**c**) first-order derivative model using data from 2011, and (**d**) second-order derivative model using data from 2011.

The *in situ* spectra of 2005 and 2011 were used to calculate the Chl-a directly using the above first- and second-order derivative models. The comparison results between the estimated and measured Chl-a are shown in Figure 5.

The RMSEs validated using the first-order derivative model in 2005 and 2011 were 15.21 mg/m^3^ and 5.85 mg/m^3^, respectively. The Figure 5(a,c) show that the estimated Chl-a is consistent with the measured Chl-a, although the model slightly underestimates the Chl-a when the concentration is approximately less than 36 mg/m^3^ and overestimates higher Chl-a for both datasets. Notably, the 2011 dataset did not contain Chl-a greater than 36 mg/m^3^.

With respect to the second-order derivative model, the validation results for the 2005 dataset significantly deviated from the 1:1 curve, and the model overestimated the values for almost all samples. In contrast, the validation results for the 2011 dataset were satisfactory, and this result could also be shown by their NRMSEs. In conclusion, the validation results of the first-order derivative model were satisfactory and consistent for different datasets, and thus the first-order derivative model at 699 nm was preferably chosen for the Chl-a estimation.

**Figure 6:**
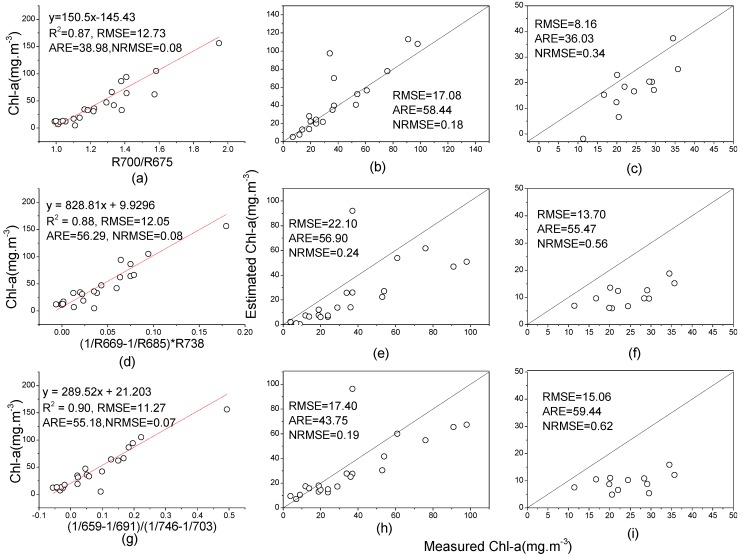
Three types of model built using the data from 2004 and their validation results using the data from 2005 and 2011. The band ratio model built using data from (**a**) 2004 and its validation using data from (**b**) 2005 and (**c**) 2011; three-band model built using data from (**d**) 2004 and its validation using data from (**e**) 2005 and (**f**) 2011; four-band model built using data from (**g**) 2004 and its validation using data from (**h**) 2005 and (**i**) 2011.

To compare the performance of the first-order derivative model with that of other models, the band ratio, three-band and four-band models were all built based on the 2004 dataset, and the results are shown in Figure 6. Additionally, to evaluate the performance of each model in other datasets, the three models were validated using the data from 2005 and 2011.

Comparing the model results of Figure 4(a), Figure 6(a,d,g) show that the first-order derivative model has a lower model accuracy (R^2^ = 0.75, RMSE = 17.84 mg/m^3^) than the other models because the latter have a higher goodness of fit (R^2^ > 0.85) and lower RMSE (RMSE < 15.00 mg/m^3^), although the ARE differs little. The first-order derivative model performed worse than the two-band, three-band and four-band models, indicating that more bands used in the model building can increase the goodness of fit.

However, the validation results (Figure 5(a,c); Figure 6(b,c,e,f,h,i)) showed an opposite tendency in the estimation precision, with more samples in validation of three models deviating from the 1:1 line (Figure 6). When validated by datasets in 2005 and 2011, the RMSEs of the first-order derivative model with 15.21 and 5.85 mg/m^3^ were much lower, whereas RMSEs of other models were greater than 17.08 and 8.16 mg/m^3^, respectively. The ARE had similar tendencies. The NRMSEs show that all of the three models for 2004 had better validation in 2005 than in 2011. The first-order derivative model performed better than the other models in the validation, showing that fewer bands in a model can help increase the validation accuracy.

To further test the validation performance of the first-order derivative model, the calibration and validation datasets were switched in turn. First, the data from 2005 were used to build the model, and the data from 2004 and 2011 were used to validate it; second, the data from 2011 were used to build the model, and the data from 2004 and 2005 were used to validate it. The results showed that the first-order derivative model had consistently better validation results than the other models.

This difference can be explained by two aspects: (1) The optical characteristics of the water at different dates may have great variations, which can be reflected in the multiple bands of the spectral curve. A combination of more bands will produce more uncertainty, and fewer bands can thus reduce such uncertainty. Therefore, although the first-order derivative model was not very satisfactory in a single dataset, the model calculated by the spectral data after smoothing suppressed the uncertainty and thus increased the model availability for new datasets. (2) More parameters in a model fit the data better but produce higher variance; fewer parameters in a model do not fit the data satisfactorily, but the goodness of fit is stable in different datasets [32]. The first-order derivative model using a single band was parsimonious and stable, and its validation results were satisfactory, in contrast with other models that produced unsatisfactory results in new datasets without model tuning. The first-order derivative model can achieve good validation results in multiple datasets, and it was thus chosen as the best model for the Chl-a estimation in the lake. 

### 3.4. Estimation of Chl-a Based on HJ1/HSI Data

HSI/HJ1 data was performed by geometric correction and 6S atmospheric correction, and then smoothed using the low pass convolution method with a kernel size of 7 pixels. The region outside Taihu Lake was masked by the boundary of the lake. The HSI bands with central wavelengths at 701.66 nm and 696.845 nm were used to calculate the first-order derivative image.

**Figure 7:**
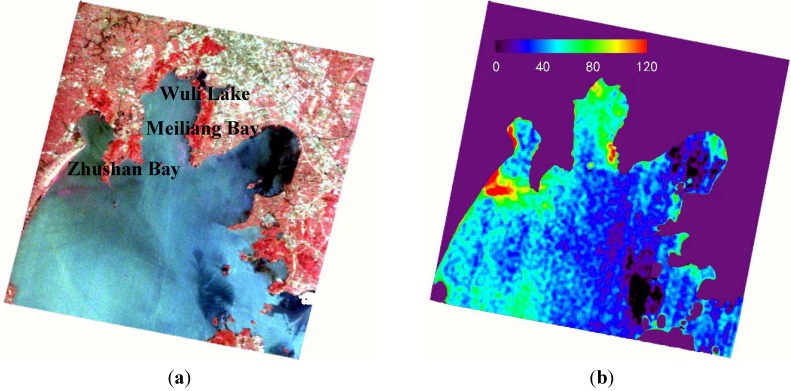
The HJ1/HSI image of Taihu Lake and the estimation result of Chl-a. (**a**) The false color composite image. (**b**) The distribution of Chl-a.

The Chl-a in the lake was calculated according to the first-order derivative model, Chl-a = 178991 × R699' + 37.766 , built in section 3, and compared with the false color composite image of 726 nm, 673 nm, and 565 nm, both results were shown in Figure 7.

Figure 7 shows that the image calculated using the first-order derivative model can generally indicate the regularity of the Chl-a distribution, although there is no ground truth value for validation. The historical data show that the Chl-a distribution in Taihu Lake had the highest content in Wuli Lake and that the Chl-a in Meiliang Bay showed a high to low trend from the bay mouth to the central lake, mainly due to the gradual reduction in the N and P nutrients from the Meiliang Bay estuary to Taihu Lake; the Chl-a in East Taihu was relatively low due to the massive growth of submerged plants [33]. The retrieved Chl-a in Figure 7(b) was concentrated in the western lake and the entrance of the Meiliang Bay, which is consistent with the previously measured water quality distribution of Taihu Lake.

The growth of algae in Taihu Lake generally involves four periods: sinking sleep (December of the prior year to February), floating recovery (March–April), massive growth (April–September) and floating accumulation (April–November) [34]. May in a year is in the early stage of cyanobacteria bloom. The algae begin to grow in May, and local algae blooms begin to emerge. The algae bloom appearing near Zhushan Bay and Meiliang Bay display pink color characteristics in the false color image (Figure 7(a)) because its spectral feature is similar to that of the plants. The Chl-a above 100 mg/m^3^ calculated indicated the bloom information around these two bays very well (Figure 7(b)), and was comparable with that retrieved in May 2009 for Taihu Lake [35]. The above results show that the spectral smoothing and derivative analysis can be used to quantitatively estimate the Chl-a distribution of HJ1 image.

## 4. Conclusions

This study applied derivative analysis to estimate the Chl-a in Taihu Lake, China, and demonstrated the necessity of spectral smoothing before building the derivative model. Three smoothing methods, *i.e.*, “mean-filter algorithm”, “Savitzky-Golay polynomial smoothing” and “kernel regression algorithm”, were calculated for comparison, and the kernel regression smoothing with a window width of 5 nm was shown to work well.

With respect to the derivative spectrum of the 2004 dataset, the first-order derivative model at 699 nm was robust and had consistently high validation accuracy in multiple datasets. The RMSE values were 15.21 mg/m^3^ and 5.85 mg/m^3^, respectively, when the model was validated using the *in situ* spectra of 2005 and 2011. The model also produced much better results than those of the band ratio, three-band and four-band models, indicating that the model with the fewest bands is parsimonious and stable and thus has better availability in practice. The Chl-a distribution calculated using the first-order derivative model based on the HSI/HJ1 remote sensing image can indicate the actual Chl-a distribution trend very well. The results demonstrated that spectral derivative analysis with kernel regression smoothing is an effective method for Chl-a estimation in water.
